# Meningitis Caused by Toscana Virus Is Associated with Strong Antiviral Response in the CNS and Altered Frequency of Blood Antigen-Presenting Cells

**DOI:** 10.3390/v7112909

**Published:** 2015-11-11

**Authors:** Stefania Varani, Francesco Gelsomino, Michele Bartoletti, Pierluigi Viale, Antonio Mastroianni, Elisabetta Briganti, Patrizia Ortolani, Francesco Albertini, Carlo Calzetti, Francesca Prati, Patrizia Cenni, Gastone Castellani, Silvia Morini, Giada Rossini, Maria Paola Landini, Vittorio Sambri

**Affiliations:** 1 Department of Experimental, Diagnostic and Specialty Medicine, University of Bologna, 40138 Bologna, Italy; mariapaola.landini@unibo.it (M.P.L.); vittorio.sambri@unibo.it (V.S.); 2 Unit of Microbiology, Regional Reference Centre for Microbiological Emergencies (CRREM), St. Orsola-Malpighi University Hospital, via Massarenti 9, 40138 Bologna, Italy; francesco.gelsomino1@gmail.com (F.G.); silvia-morini@libero.it (S.M.); giada.rossini@unibo.it (G.R.); 3 Infectious Disease Unit, Department of Medical and Surgical Science, University of Bologna, 40138 Bologna, Italy; bartolettimichele@yahoo.it (M.B.); pierluigi.viale@unibo.it (P.V.); 4 Infectious Disease Unit, G.B. Morgagni-Pierantoni Hospital, 47121 Forlì, Italy; antoniomastroianni@yahoo.it (A.M.); elisabettabriganti@libero.it (E.B.); 5 Infectious Disease Unit, Infermi Hospital, 47923 Rimini, Italy; o.patrizia@tiscali.it; 6 Division of Infectious Diseases, St. Maria delle Croci Hospital, 48121 Ravenna, Italy; fr.albertini@libero.it; 7 Unit of Infectious Diseases and Hepatology, Parma University Hospital, 43126 Parma, Italy; ccalzetti@ao.pr.it; 8 Infectious Disease Division, Reggio Emilia Hospital, 42100 Reggio Emilia, Italy; francesca.prati2@asmn.re.it; 9 Emergency Department, Ospedale Civile St. Maria della Scaletta, 40026 Imola, Italy; p.cenni@ausl.imola.bo.it; 10 Department of Physics and Astronomy and Galvani Center for Biocomplexity, University of Bologna, 40127 Bologna, Italy; gastone.castellani@unibo.it; 11 Unit of Microbiology, “AUSL della Romagna” Hub Laboratory, 47522 Pievesestina, (FC), Italy

**Keywords:** Toscana virus, viral meningitis, monocytes, dendritic cells, cytokines

## Abstract

Toscana virus (TOSV) is a Phlebotomus-transmitted RNA virus and a frequent cause of human meningitis and meningoencephalitis in Southern Europe during the summer season. While evidence for TOSV-related central nervous system (CNS) cases is increasing, little is known about the host defenses against TOSV. We evaluated innate immune response to TOSV by analyzing frequency and activation of blood antigen-presenting cells (APCs) and cytokine levels in plasma and cerebrospinal fluid (CSF) from patients with TOSV neuroinvasive infection and controls. An altered frequency of different blood APC subsets was observed in TOSV-infected patients, with signs of monocytic deactivation. Nevertheless, a proper or even increased responsiveness of toll-like receptor 3 and 7/8 was observed in blood APCs of these patients as compared to healthy controls. Systemic levels of cytokines remained low in TOSV-infected patients, while levels of anti-inflammatory and antiviral mediators were significantly higher in CSF from TOSV-infected patients as compared to patients with other infectious and noninfectious neurological diseases. Thus, the early host response to TOSV appears effective for viral clearance, by proper response to TLR3 and TLR7/8 agonists in peripheral blood and by a strong and selective antiviral and anti-inflammatory response in the CNS.

## 1. Introduction

Toscana virus (TOSV) is a Phlebotomus-borne virus, classified within the sandfly fever virus group of the genus *Phlebovirus* (family *Bunyaviridae*). Three other major serotypes, Sicilian, Naples and Cyprus, belong to the sandfly fever virus group, but only TOSV exhibits tropism for the central nervous system (CNS) [1]. In fact, TOSV can cause aseptic meningitis, meningoencephalitis or encephalitis, which exhibit better clinical outcome compared to other neuro-arbovirosis, such as West Nile virus (WNV) infection [2]. Although self-resolving in most cases, TOSV infection of the CNS may be severe in some patients who need intensive care or may experience ischemic complications or hydrocephalus [3,4,5,6].

In the European Mediterranean countries, including Italy, Spain, Portugal, France, Greece and Cyprus, TOSV is one of the most frequent causes of CNS infection during the summer season [7]. Recent evidence indicates TOSV infection as emergent in Northeast Italy [8,9,10]. Furthermore, a growing number of TOSV infections have been reported in travelers returning from the Mediterranean basin to other European countries and the US [2].

Despite the fact that evidence of TOSV-related CNS cases is increasing, little is known about early host defense against this infection. During acute viral infection, cells of the innate immune system are the first to be activated. Among these, dendritic cells (DCs) and monocytes are professional antigen-presenting cells (APCs) and are key players in immune responses by recognizing conserved microbial patterns through toll-like receptors (TLRs), engulfing pathogens, and activating and orchestrating T-cell responses [11].

Distinct subsets of circulating DCs can be identified in the peripheral blood, including CD1c/BDCA-1^+^ and CD141/BDCA-3^+^ myeloid dendritic cells (mDCs) and CD303/BDCA-2^+^ plasmacytoid dendritic cells (pDCs); mDCs and pDCs recognize RNA viruses by TLR3 and TLR7, respectively [11]. In addition, monocytes can be subdivided into three subsets based on the expression of CD14 and CD16 markers; the classical monocytes are CD14^++^ and do not express CD16, the intermediate monocytes are CD14^++^ and CD16^+^, and the nonclassical monocytes express low levels of CD14 together with high CD16 (CD14^+^CD16^++^) [11] and exhibit high levels of TLR7/8 to detect virally infected cells [12].

Recent evidence indicates that phleboviruses, including TOSV, are capable to infect cells that express DC-SIGN, such as mDCs [13], thus suggesting that mDCs may be target cells in the pathogenesis of these infections. Nevertheless, many aspects of TOSV infection and pathogenesis remain unresolved, including its interactions with the host immune system.

This study aimed to evaluate innate responses in patients with TOSV neuroinvasive infection following the hypothesis that such responses would be potent and efficient during TOSV infection. In fact, TOSV generally causes a benign and short-course meningitis, which implies the activation of early and effective host responses. We designed the study as follows (1) to evaluate the frequency and activation status of blood APCs; (2) to evaluate the functional status of TLRs mainly expressed by APCs and involved in the response to RNA viruses; and (3) to quantify cytokine levels in the plasma and cerebrospinal fluid (CSF) of patients with neuroinvasive infection caused by TOSV as compared to healthy controls and/or patients with other infectious and noninfectious neurological diseases.

## 2. Results

### 2.1. Patient Characteristics

Twenty-six patients suffering from meningitis or meningoencephalitis caused by TOSV were included in the study (Table 1). The age of the patients ranged from 18 to 80 years, with a median age of 47 and an interquartile range (IQR) of 40–64 years. The female-to-male ratio was 7/19. CSF data were available from 22 patients, and all 22 had elevated CSF protein concentration, with a mean CSF protein concentration of 117 mg/dL and an IQR of 96–170 mg/dL (normal range: 20–40 mg/dL). Furthermore, 16 of the 22 patients had pleocytosis with mononuclear cell prevalence. The median WBC count was 300 cells/mm^3^ with an IQR of 149–404 cells/mm^3^. A combination of elevated CSF protein concentration and elevated peripheral WBC count was observed in 16 patients.

The clinical course of TOSV infection was mild with classical aseptic meningitis in 17 out of 26 cases. One case presented with headache and fever only, while 8 additional patients experienced meningoencephalitis. Two meningoencephalitis patients developed an altered mental status and required intensive care. All but one of the TOSV-positive cases showed a complete recovery when discharged from the hospital; one patient exhibited paresthesia of the extremities that lasted for seven months.

**Table 1:** Demographic variables, clinical data, cerebrospinal fluid characteristics and outcome of patients with TOSV infection. Total Patients *n* = 26.

	*n*	Median (IQR)	Percentage (%)
**Demographic factors**			
Age		47 (40–64)	
Male sex	19		73.1
**Comorbidities**			
Charlson score		0 (0–0.5)	
**Clinical characteristics of TOSV infection**			
Meningitis	17		65.4
Meningoencephalitis	8		30.8
Fever without CNS involvement	1		3.8
**CSF characteristics**			
WB cells (cells/mm^3^)		300 (149–404)	
Protein content (mg/dL)		117 (96–170)	
Glucose level (mg/dL)		58 (48–65)	
CSF glucose/serum ratio		0.6 (0.5–0.7)	
**Outcome ^a^**			
ICU admission ^b^	2		8.0
Complete resolution	23		92.0
Long term sequelae	0		0

**Notes:** TOSV, Toscana virus; IQR, interquartile range; CSF, cerebrospinal fluid; CNS, central nervous system; WB, white blood; ICU, intensive care unit; ^a^ data not available for one patient; ^b^ Two patients presented with meningoencephalitis and altered mental status; *n*, means number.

### 2.2. Changes of the Blood APC Composition in TOSV-Infected Patients

**Figure 1:**
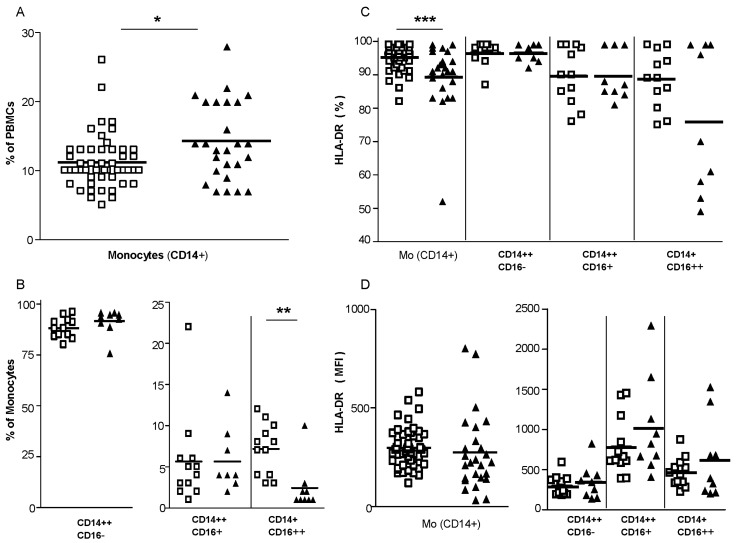
Changes in blood monocytes during TOSV infection. Peripheral blood mononuclear cells (PBMCs) from the study participants were stained with monoclonal antibodies for subsequent flow cytometric analyses. Monocytes (CD14^+^) were gated as described in Materials and Methods. Cell frequencies (**A**,**B**) and human leukocyte antigen (HLA)-DR expression levels (**C**,**D**) of the circulating monocytes are reported. *N* = 26 TOSV+ patients and *n* = 49 healthy controls were evaluated for total monocytes, while *n* = 9 TOSV+ patients and *n* = 12 healthy donors were analyzed for monocytic subsets. White squares indicate healthy controls and black triangles indicate TOSV-infected patients. MFI, mean fluorescence intensity. Data were analyzed using the non-parametric Mann–Whitney *U* test. * indicates *p* ≤ 0.05, ** indicates *p* ≤ 0.01 and *** indicates *p* ≤ 0.001.

**Figure 2:**
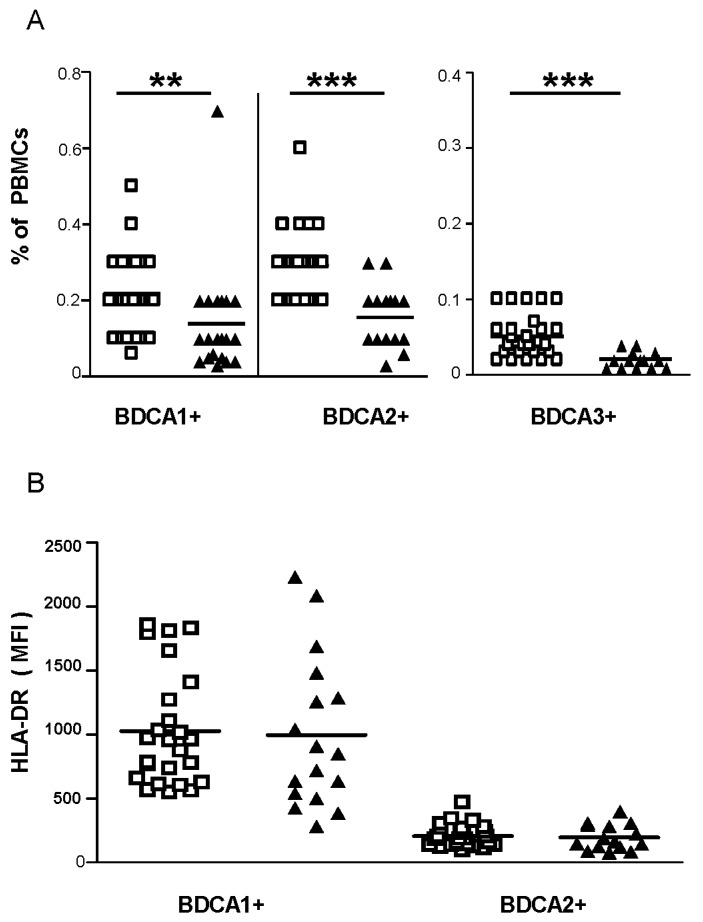
Cell frequencies and activation markers of circulating DCs (BDCA-1^+^, BDCA-2^+^, and BDCA-3^+^). Peripheral blood mononuclear cells (PBMCs) from the study participants were stained with monoclonal antibodies for subsequent flow cytometric analyses. mDCs (BDCA-1^+^ and BDCA-3^+^ DCs) as well as pDCs (BDCA-2^+^ DCs) were gated as described in Materials and Methods. Lower frequencies of circulating DCs were observed in TOSV-infected patients (**A**) with no difference in the expression levels of human leukocyte antigen (HLA) class II molecules compared to healthy controls (**B**). White squares indicate healthy controls and black triangles indicate TOSV-infected patients. *N* = 21 TOSV+ patients and *n* = 26 healthy controls were analyzed. MFI, mean fluorescence intensity. Data were analyzed using the non-parametric Mann–Whitney *U* test. ** indicates *p* ≤ 0.01 and *** indicates *p* ≤ 0.001.

We observed an increased frequency of total blood monocytes in TOSV-infected patients compared to healthy controls (Figure 1A). By examining the different monocytic subsets in nine TOSV-infected patients and 12 healthy controls, we observed that the proportion of the distinct subsets of monocytes was altered with the frequency of nonclassical monocytes being reduced in TOSV-infected patients when compared to healthy controls (Figure 1B). Furthermore, monocytes from TOSV-infected patients exhibited a significant reduction in the percentage of cells expressing the major histocompatibility complex (MHC) class-II molecules compared to healthy controls (Figure 1C), while expression levels of MHC class II molecules did not differ on monocytes obtained from patients and healthy controls (Figure 1D). In TOSV-infected patients, the decline in MHC-II expressing cells appeared more evident in the nonclassical monocytes subset. Conversely, a lower frequency of blood BDCA-1^+^, BDCA-2^+^ and BDCA-3^+^ DCs was observed in TOSV-infected patients (Figure 2A) with no difference in the percentage of MHC class-II positive cells (data not shown) nor in the MHC class-II expression levels (Figure 2B) when compared to healthy controls.

### 2.3. Responsiveness of TLR3 and TLR7/8 in *ex Vivo* Stimulation Assay of Blood Cells from TOSV-Infected Patients

**Figure 3:**
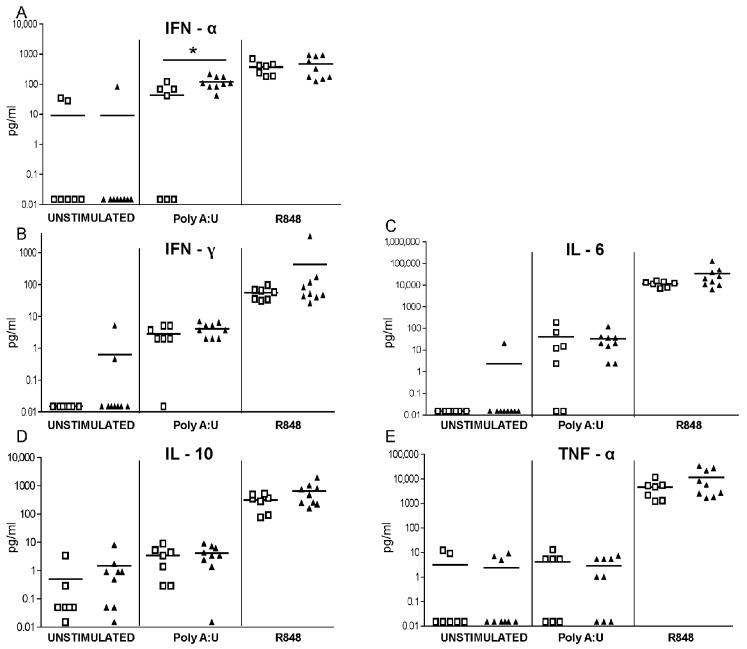
TLR-induced cytokine responses in blood cells from TOSV-infected patients and healthy controls. Blood cells were left unstimulated or stimulated with poly(A:U) (TLR3 ligand; 20 µg/mL) or Resiquimod-R848 (TLR7/8 ligand; 10 µg/mL). After 24 h, supernatants were collected and analyzed for cytokine levels. White squares indicate healthy controls and black triangles indicate TOSV-infected patients. *N* = 8 TOSV-infected patients and *n* = 7 healthy controls were evaluated. Data were analyzed using the non-parametric Mann–Whitney *U* test. * indicates *p* ≤ 0.05.

To evaluate the functional status of TLRs expressed by APCs and involved in responses to RNA viruses, we stimulated whole blood samples from eight TOSV-infected patients and seven healthy controls with TLR3 or TLR7/8 agonists for 24 h, after which the supernatants were collected for cytokine assays. The production of soluble factors by agonist-stimulated cells was higher than the amounts spontaneously secreted by unstimulated blood samples, especially for the TLR7/8-agonist R848 (Figure 3A–E). No significant differences were observed in unstimulated blood cells and in TLR7/8-mediated cytokine responses between TOSV-positive patients and healthy controls, while cells from TOSV-positive patients exhibited an increased release of IFN-α in response to the TLR3 ligand poly(A:U) as compared to healthy controls.

### 2.4. Cytokine Levels in the Plasma and CSF of TOSV-Infected Patients

**Figure 4:**
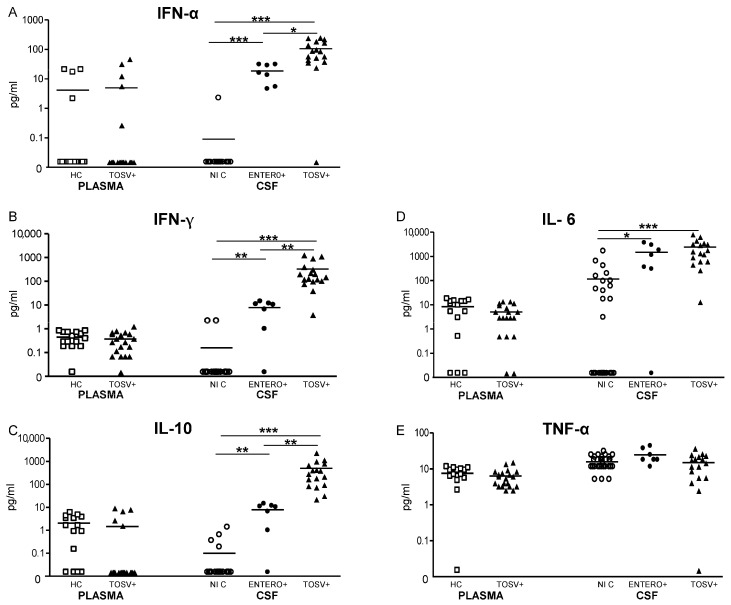
Levels of pro-inflammatory and anti-inflammatory cytokines in plasma and CSF from TOSV-infected patients and controls. The levels of IFN-α, IFN-γ, IL-6, IL-10, and TNF-α (**A**–**E**) were measured in the plasma samples from TOSV-infected patients (TOSV+) and from healthy controls (HC) as described in Materials and Methods. Cytokine levels were also measured in the CSF samples from TOSV-infected patients (TOSV+) and from patients with enterovirus meningitis (ENTERO+) and with noninfectious diseases of the CNS (noninfectious controls, NIC). White squares designate healthy controls (*n* = 15), white circles indicate noninfectious controls (*n* = 30), black circles designate enterovirus meningitis (*n* = 7) and black triangles indicate TOSV-infected patients (*n* = 17, 11 patients with meningitis and six patients with meningoencephalitis). Data were analyzed using the non-parametric Mann–Whitney *U* test. * indicates *p* ≤ 0.05, ** indicates *p* ≤ 0.01 and *** indicates *p* ≤ 0.001.

The levels of IL-6, IL-10, TNF-α, IFN-α and IFN-γ were examined in the plasma samples from 17 infected patients and 15 healthy controls (Figure 4). We did not observe any difference in the plasma levels of these cytokines between TOSV-infected patients and controls. Then, we compared cytokines levels in the CSF samples from TOSV infected patients (*n* = 17) with those obtained from two control groups, *i.e.*, CSF samples obtained from patients suffering of noninfectious diseases of the CNS (*n* = 30) and from patients with enterovirus meningitis (*n* = 7). The levels of IL-6, IL-10, IFN-α and IFN-γ were significantly higher in the CSF samples from patients suffering of viral meningitis or meningoencephalitis as compared to noninfectious CNS diseases. While no differences were observed in the intrathecal levels of IL-6 between patients infected with TOSV and enterovirus, CSF levels of IFN-α, IFN-γ and IL-10 were significantly higher in TOSV-infected patients as compared with enterovirus-infected patients. Among TOSV-infected patients, no difference was observed in CSF cytokine production between patients with meningitis and patients with meningoencephalitis.

## 3. Discussion

Fluctuation in blood DC numbers has been observed in viral infections; acute, short-lasting infections such as those caused by dengue virus and influenza virus are associated with an expansion of blood DC, while viruses inducing chronic/persistent infection (HIV, hepatitis C virus, human cytomegalovirus) mainly cause a decline of circulating DC number/frequency [14,15]. Our study examines the innate immune response in patients suffering from TOSV infection. Despite causing an acute and fast-resolving infection, TOSV was associated with a significant reduction of mDC and pDC frequency in infected patients. Whether this was due to infection of DCs, ongoing apoptosis or to a diminished re-population of blood cells from the bone marrow remains speculative.

On other hand, the frequency of blood monocytes was significantly increased in TOSV-infected patients compared to healthy controls; this phenomenon was restricted to classical and intermediate monocytes, resembling the “inflammatory” monocytes that have been described in mice [16], as the proportion of nonclassical monocytes was reduced in infected patients. Monocyte infiltration is a hallmark of CNS inflammation, including when caused by viral infections [17]. Evidence indicates that CNS inflammation provokes bone marrow monocyte responses by inducing emigration of existing bone marrow CCR2-positive monocytes into the circulation, and/or by generation of new monocytes in the bone marrow in experimental models of neuroinvasive infections [16,18]. These mechanisms may also apply to TOSV-induced neuroinflammation.

Even though TOSV-infected patients had increased percentage of circulating monocytes, these cells displayed an impaired phenotype with a decline of cells expressing MHC class-II molecules. As MHC class-II downregulation is considered a surrogate marker for monocyte dysfunction [19], monocytic deactivation may lead to reduced T-cell activity, thus facilitating viral replication and worsening the TOSV infection. In support of this hypothesis, we observed a profound deactivation of blood monocytes in the two patients who required ICU admission (not shown). However, due to the small study group, we could not perform a correlation analysis between the alteration of monocytic phenotype and the severity of the TOSV infection.

Despite blood monocyte deactivation, APC-mediated responses to single stranded RNA (ssRNA) did not appear to be altered in TOSV-infected patients, as TLR7/8 responsiveness to the specific ligand R848 was equivalent in TOSV-infected patients and healthy controls. Further, stimulation of TLR3—a sensor for double stranded RNA (dsRNA)—induced a higher release of IFN-α in blood cells from TOSV-infected patients as compared to healthy controls. This finding parallels the high IFN-α levels that were observed in the CSF of TOSV-infected patients and suggests an involvement of TLR3 in the antiviral response to TOSV.

Cytokines are produced intrathecally in patients with viral meningitis or meningoencephalitis, and different viruses produce unique inflammatory responses *via* different pathogenic pathways [20]. While systemic levels of cytokines remained low in TOSV-infected patients, intrathecal levels of pro-inflammatory, anti-inflammatory and antiviral mediators were found increased, indicating local production within the CNS. When comparing levels of cytokines in CSF samples from patients with TOSV infection to those from patients with enterovirus meningitis, we observed that TOSV induced higher intrathecal production of IFN-α, IFN-γ and IL-10, which suggests the activation of a selective antiviral and anti-inflammatory response in the CNS of TOSV-infected patients. As viral entry and dissemination as well as viral structure differ significantly between TOSV and enteroviruses, neuropathogenesis of these viral infections is likely dissimilar, which may explain variation in cytokine levels that we observed in the CSF of infected patients. We observed a stronger immune response in the CNS of TOSV-infected patients when compared to enterovirus-infected patients; this is in line with observations in other neuroinvasive arbovirosis, such as those caused by flaviviruses, where an intense immune response in the CNS is often found [21].

The IFN system represents the first line of host defense against neuroinvasive virus infection [22] and is crucial to generate an antiviral state of brain cells and cytotoxic defense to neurotropic viruses [23,24]. Recent evidence indicates that TOSV is susceptible to type-I IFN as its replication is inhibited by IFN-β [25]; indeed an important role for the IFN system in the antiviral response against TOSV has been demonstrated by the ability of TOSV nonstructural protein to inhibit type-I IFN release [26,27].

Further, high expression of IL-10 is beneficial during the course of CNS inflammatory diseases by promoting survival of neurons and glial cells in the brain; progressively decreasing levels of intrathecal IL-10 are associated to severe symptoms in patients with neuroinvasive tick-borne encephalitis virus (TBEV) [20] and in an experimental model of Japanese encephalitis virus (JEV) infection [28]. Reduced levels of IL-10 also allow amplification of pro-inflammatory Th1 cytokine cascade, which leads to brain damage, as shown in post-mortem studies and in experimental models of neuro-arbovirosis, including WNV [29,30,31], JEV [32,33] and TBE [34,35] infections. The antiviral response to TOSV in the CNS appeared to be properly modulated by the IL-10-mediated anti-inflammatory counterpart and all TOSV-infected patients in our study group cleared the neuroinvasive infection without permanent damage. Conversely, low TNF-α levels were found in the CSF of the same patients, as shown in meningitis caused by other viruses [20,36].

In conclusion, the early host response to TOSV appears effective for viral clearance, by proper activity in response to TLR3 and TLR7/8 agonists in the periphery and by a strong mixed antiviral and anti-inflammatory response in the CNS. These results provide a better understanding of the innate host response to TOSV, which could contribute to identification of phenotypic risk factors of neurologic complications and for the development of appropriate treatment.

## 4. Materials and Methods

### 4.1. Study Population

During the summer season 2012–2013, 26 adult patients with meningitis or meningoencephalitis admitted to healthcare facilities in the Emilia-Romagna region (Northern Italy) and diagnosed with active TOSV infection were included in the study. Laboratory diagnosis of acute TOSV infection was performed at the Regional Reference Centre for Microbiological Emergencies (Unit of Microbiology, St. Orsola-Malpighi University Hospital, Bologna, Italy). Peripheral blood and CSF samples were collected during the acute phase of the disease (*i.e.*, 1–5 days after the onset of symptoms) as part of the diagnostic routine. TOSV diagnosis was performed by serologic and molecular methods as reported [10]. Twenty-two patients tested positive both for TOSV RNA in CSF and for anti-TOSV IgG and/or IgM in the corresponding serum/plasma sample, 2 patients tested positive for TOSV RNA in CSF and negative for specific antibodies in serum/plasma and the 2 remaining patients tested positive only for anti-TOSV IgG and IgM in serum (CSF was not available in one case and tested negative for TOSV RNA in the other case). As healthy controls, blood samples from 49 blood donors were obtained from the Blood Bank, St. Orsola-Malpighi University Hospital, Bologna, Italy. Age of healthy controls ranged from 26 to 67 years, with a median age of 44, female-to-male ratio was 17/32. As additional control groups, CSF samples were obtained from patients suffering of enterovirus meningitis (*n* = 7, age range 1–36 years, median age: 25, female-to-male ratio: 1/6) and of noninfectious CNS diseases including brain cancer, trauma and demyelinating disorders (*n* = 30, age range 19–89 years, median age: 54, female-to-male ratio: 14/16). All blood donors and non-healthy controls were resident in the Emilia-Romagna region. As TOSV serostatus is not routinely tested by the Blood Bank, we performed serological assays to determine anti-TOSV IgG and IgM in plasma from 49 blood donors included as healthy controls. We also tested CSF from non-healthy controls (7 patients with enterovirus meningitis and 30 patients with noninfectious CNS diseases) for anti-TOSV antibody. Plasma and CSF samples were analyzed by indirect immunofluorescence test (*Anti-sandfly fever virus Mosaic*, Euroimmun, Lübeck, Germany). No IgM was detected in plasma or CSF samples from blood donors and non-healthy controls, thus excluding active TOSV infection in the control groups, while 3 individuals exhibited anti-TOSV IgG, as a sign of a past infection.

The study was conducted in accordance with the Declaration of Helsinki, and the protocol was approved by the Ethics Committee of the St. Orsola Malpighi University Hospital (project identification code: 91/2011/U/Tess). Written informed consent was obtained from all patients. Clinical assessments were completed at hospital admission and discharge, according to the study protocol. CSF evaluation included a standard cytochemical analysis with the measurement of protein and glucose concentration and white blood cell (WBC) count. A CSF WBC count was considered altered when the number of cells was over 6 cells/mm^3^.

### 4.2. Blood Collection and Cell Preparation

Six to eight milliliters of peripheral blood were drawn from patients and healthy controls, collected into K-EDTA tubes and processed for this study within 30 h from collection. Samples were centrifuged, and the plasma was removed and stored at −80 °C for analyzing cytokines levels. Subsequently, the original volume of each blood sample was reconstituted with RPMI medium and employed for immunophenotyping of the peripheral blood mononuclear cells.

### 4.3. Immunophenotyping

FcR blocking reagent (Miltenyi Biotec, GmbH, Germany) and mouse monoclonal antibodies conjugated with fluorescein isothiocyanate (FITC), phycoerythrin (PE), or peridinin chlorophyll protein (PerCP) were added. Antibodies used for cell-surface staining included the B-cell marker CD19 (clone SJ25C1, eBioscience, San Diego, CA, USA); the monocyte markers CD14 and CD16 (clones TUK4 and VEP13, respectively, Miltenyi Biotec); DC markers, including blood dendritic cell antigen (BDCA)-1, BDCA-2, and BDCA-3 (clones AD5-8E7, AC144 and AD5-14H12, respectively, Miltenyi Biotec), and the activation marker HLA-DR (clones AC122, Miltenyi Biotec). Subpopulations of monocytes were defined by the gating strategy suggested by Heimbeck *et al.* [37]. Briefly, monocytes were defined by sequential gating in light scatter plots and in CD14/HLA-DR dot plot before segregating monocyte subsets in CD14/CD16 dot plot. Subpopulations of DCs were defined by the absence of lineage markers for B lymphocytes and monocytes and the expression of BDCA-1/BDCA-3 or BDCA-2 for mDCs and pDCs, respectively. After staining, to ensure an optimal lysis of erythrocytes, the Red Blood Cell Lysis Solution (Miltenyi Biotec) was utilized. Cells were then immediately acquired using a FACS Calibur System (Automated Flow Cytometer, BD Biosciences, San Diego, CA, USA) and analyzed using the BD CellQuest Pro Software (Becton Dickinson, NJ, USA).

### 4.4. Stimulation of Blood Cells with Specific TLR Ligands

Whole blood samples were diluted 1:1 with RPMI medium and separate aliquots (200 µL) of diluted blood distributed into a 96-well flat bottom plate for cell cultures were left unstimulated or stimulated either with poly(A:U) (TLR3 ligand; 20 µg/mL; InvivoGen, San Diego, CA, USA) or Resiquimod-R848 (TLR 7/8 ligand; 10 µg/mL, InvivoGen). After 24 h of incubation at 37 °C in 5% CO_2_, culture supernatants were collected by centrifugation and stored at −80 °C for cytokine determination. The different TLR ligands used in these assays exert their effects on distinct immune cell subsets: poly(A:U) interacts with TLR3 expressed within mDCs [38], Resiquimod-R848 activates TLR7 and TLR8, with TLR7 being expressed mainly within pDCs and B cells [39], whereas TLR8 is found within monocytes and mDCs [12,40]. The choice of stimulants reflects a desire, based on our own knowledge and experience [41] to focus on APC-mediated response to RNA viruses.

### 4.5. Determination of Cytokines Levels in Supernatants, Plasma and CSF Samples

The levels (pg/mL) of IL-6, IL-10, IFN-α, IFN-γ and TNF-α were measured in blood cell supernatants, plasma and CSF samples by employing the Procarta Immunoassay Kit with magnetic beads (Affymetrix Inc., eBioscience, San Diego, CA, USA). This Luminex-based method utilizes microspheres as the solid support for a conventional sandwich immunoassay to simultaneously measure multiple cytokines. Following the manufacturer’s instructions, anti-cytokine antibody-coupled beads were added to each well of a 96-well plate. After washing, standards and samples (diluted 1:10 in appropriate diluents) were then pipetted into each well and incubated for 120 min at room temperature on a plate shaker. Following 3 washes, a biotinylated detector antibody was added to each well and the plate was incubated for 30 min in the dark. After washing, a final incubation with Streptavidin-PE was performed. Finally, complexes were resuspended in 120 µL of detection buffer, and 100 beads were counted during acquisition on the Luminex^®^ 200™ instrument (Luminex Corporation, Austin, TX, USA). The mean fluorescence intensity was analyzed and the concentration of cytokines in the supernatants, plasma or CSF was calculated in pg/mL. The assay sensitivity was 0.4 pg/mL for IL-6, 0.15 pg/mL for IL-10, 2.25 pg/mL for IFN-α, 0.1 pg/mL for IFN-γ, and 0.26 pg/mL for TNF-α.

### 4.6. Statistical Analysis

Categorical variables (gender, clinical characteristics and disease outcome) were reported as absolute values and relative frequencies. Continuous variables (age, CSF characteristics, *etc.*) were reported as medians with IQRs. Due to the nonparametricity of the experimental data, the Mann–Whitney *U* test was employed to evaluate significance between two unpaired groups of data for the FACS immunophenotyping and cytokine levels (TOSV-positive patients *vs.* healthy controls). A *p*-value of 0.05 or less was considered significant. Data analysis was performed using R (R Foundation for Statistical Computing c/o Institute for Statistics and Mathematics Wirtschaftsuniversitat Wien, Austria) and Mathematica (developed by Wolfram Research of Champaign, IL, USA).

## References

[1] Cusi M.G., Savellini G.G., Zanelli G. (2010). Toscana virus epidemiology: From Italy to beyond. Open Virol. J..

[2] Jaijakul S., Arias C.A., Hossain M., Arduino R.C., Wootton S.H., Hasbun R. (2012). Toscana meningoencephalitis: A comparison to other viral central nervous system infections. J. Clin. Virol..

[3] Baldelli F., Ciufolini M.G., Francisci D., Marchi A., Venturi G., Fiorentini C., Luchetta M.L., Bruto L., Pauluzzi S. (2004). Unusual presentation of life-threatening Toscana virus meningoencephalitis. Clin. Infect. Dis..

[4] Bartels S., de Boni L., Kretzschmar H.A., Heckmann J.G. (2012). Lethal encephalitis caused by the Toscana virus in an elderly patient. J. Neurol..

[5] Kuhn J., Bewermeyer H., Hartmann-Klosterkoetter U., Emmerich P., Schilling S., Valassina M. (2005). Toscana virus causing severe meningoencephalitis in an elderly traveller. J. Neurol. Neurosurg. Psychiatry.

[6] Sanbonmatsu-Gamez S., Perez-Ruiz M., Palop-Borras B., Navarro-Mari J.M. (2009). Unusual manifestation of Toscana virus infection, Spain. Emerg. Infect. Dis..

[7] Charrel R.N., Gallian P., Navarro-Mari J.M., Nicoletti L., Papa A., Sanchez-Seco M.P., Tenorio A., de Lamballerie X. (2005). Emergence of Toscana virus in Europe. Emerg. Infect. Dis..

[8] Calzolari M., Angelini P., Finarelli A.C., Cagarelli R., Bellini R., Albieri A., Bonilauri P., Cavrini F., Tamba M., Dottori M. (2014). Human and entomological surveillance of Toscana virus in the Emilia-Romagna region, Italy, 2010 to 2012. Eurosurveillance.

[9] Vocale C., Bartoletti M., Rossini G., Macini P., Pascucci M.G., Mori F., Tampieri A., Lenzi T., Pavoni M., Giorgi C. (2012). Toscana virus infections in northern Italy: Laboratory and clinical evaluation. Vector Borne Zoonotic Dis..

[10] Pierro A., Landini M.P., Gaibani P., Rossini G., Vocale C., Finarelli A.C., Cagarelli R., Sambri V., Varani S. (2014). A model of laboratory surveillance for neuro-arbovirosis applied during 2012 in the Emilia-Romagna region, Italy. Clin. Microbiol. Infect..

[11] Ziegler-Heitbrock L., Ancuta P., Crowe S., Dalod M., Grau V., Hart D.N., Leenen P.J., Liu Y.J., MacPherson G., Randolph G.J. (2010). Nomenclature of monocytes and dendritic cells in blood. Blood.

[12] Cros J., Cagnard N., Woollard K., Patey N., Zhang S.Y., Senechal B., Puel A., Biswas S.K., Moshous D., Picard C. (2010). Human CD14dim monocytes patrol and sense nucleic acids and viruses via TLR7 and TLR8 receptors. Immunity.

[13] Lozach P.Y., Kühbacher A., Meier R., Mancini R., Bitto D., Bouloy M., Helenius A. (2011). DC-SIGN as a receptor for phleboviruses. Cell Host Microbe.

[14] Miles B., Abdel-Ghaffar K.A., Gamal A.Y., Baban B., Cutler C.W. (2014). Blood dendritic cells: “Canary in the coal mine” to predict chronic inflammatory disease?. Front. Microbiol..

[15] Varani S., Rossini G., Mastroianni A., Tammik C., Frascaroli G., Landini M.P., Castellani G., Söderberg-Nauclér C. (2012). High TNF-alpha and IL-8 levels predict low blood dendritic cell counts in primary cytomegalovirus infection. J. Clin. Virol..

[16] Shi C., Pamer E.G. (2011). Monocyte recruitment during infection and inflammation. Nat. Rev. Immunol..

[17] Terry R.L., Getts D.R., Deffrasnes C., van Vreden C., Campbell I.L., King N.J. (2012). Inflammatory monocytes and the pathogenesis of viral encephalitis. J. Neuroinflamm..

[18] Ashhurst T.M., Vreden C.V., Niewold P., King N.J. (2014). The plasticity of inflammatory monocyte responses to the inflamed central nervous system. Cell. Immunol..

[19] Monneret G., Venet F., Pachot A., Lepape A. (2008). Monitoring immune dysfunctions in the septic patient: A new skin for the old ceremony. Mol. Med..

[20] Günther G., Haglund M., Lindquist L., Forsgren M., Andersson J., Andersson B., Sköldenberg B. (2011). Tick-borne encephalitis is associated with low levels of interleukin-10 in cerebrospinal fluid. Infect. Ecol. Epidemiol..

[21] Swanson P.A., McGavern D.B. (2015). Viral diseases of the central nervous system. Curr. Opin. Virol..

[22] Detje C.N., Meyer T., Schmidt H., Kreuz D., Rose J.K., Bechmann I., Prinz M., Kalinke U. (2009). Local type I IFN receptor signaling protects against virus spread within the central nervous system. J. Immunol..

[23] Weber E., Finsterbusch K., Lindquist R., Nair S., Lienenklaus S., Gekara N.O., Janik D., Weiss S., Kalinke U., Överby A.K. (2014). Type I interferon protects mice from fatal neurotropic infection with Langat virus by systemic and local antiviral responses. J. Virol..

[24] Samuel M.A., Diamond M.S. (2005). Alpha/beta interferon protects against lethal West Nile virus infection by restricting cellular tropism and enhancing neuronal survival. J. Virol..

[25] Brisbarre N.M., Plumet S., de Micco P., Leparc-Goffart I., Emonet S.F. (2013). Toscana virus inhibits the interferon beta response in cell cultures. Virology.

[26] Gori-Savellini G., Valentini M., Cusi M.G. (2013). Toscana virus NSs protein inhibits the induction of type I interferon by interacting with RIG-I. J. Virol..

[27] Gori Savellini G., Weber F., Terrosi C., Habjan M., Martorelli B., Cusi M.G. (2011). Toscana virus induces interferon although its NSs protein reveals antagonistic activity. J. Gen. Virol..

[28] Swarup V., Ghosh J., Duseja R., Ghosh S., Basu A. (2007). Japanese encephalitis virus infection decrease endogenous IL-10 production: Correlation with microglial activation and neuronal death. Neurosci. Lett..

[29] Wang Y., Lobigs M., Lee E., Mullbacher A. (2003). CD8^+^ T cells mediate recovery and immunopathology in West Nile virus encephalitis. J. Virol..

[30] Armah H.B., Wang G., Omalu B.I., Tesh R.B., Gyure K.A., Chute D.J., Smith R.D., Dulai P., Vinters H.V., Kleinschmidt-DeMasters B.K. (2007). Systemic distribution of West Nile virus infection: Postmortem immunohistochemical study of six cases. Brain Pathol..

[31] Omalu B.I., Shakir A.A., Wang G., Lipkin W.I., Wiley C.A. (2003). Fatal fulminant pan-meningo-polioencephalitis due to West Nile virus. Brain Pathol..

[32] Biswas S.M., Kar S., Singh R., Chakraborty D., Vipat V., Raut C.G., Mishra A.C., Gore M.M., Ghosh D. (2010). Immunomodulatory cytokines determine the outcome of Japanese encephalitis virus infection in mice. J. Med. Virol..

[33] Johnson R.T., Burke D.S., Elwell M., Leake C.J., Nisalak A., Hoke C.H., Lorsomrudee W. (1985). Japanese encephalitis: Immunocytochemical studies of viral antigen and inflammatory cells in fatal cases. Ann. Neurol..

[34] Gelpi E., Preusser M., Laggner U., Garzuly F., Holzmann H., Heinz F.X., Budka H. (2006). Inflammatory response in human tick-borne encephalitis: Analysis of postmortem brain tissue. J. Neurovirol..

[35] Růzek D., Salát J., Palus M., Gritsun T.S., Gould E.A., Dyková I., Skallová A., Jelínek J., Kopecký J., Grubhoffer L. (2009). CD8^+^ T-cells mediate immunopathology in tick-borne encephalitis. Virology.

[36] Glimåker M., Kragsbjerg P., Forsgren M., Olcén P. (1993). Tumor necrosis factor-α (TNF α) in cerebrospinal fluid from patients with meningitis of different etiologies: High levels of TNF alpha indicate bacterial meningitis. J. Infect. Dis..

[37] Heimbeck I., Hofer T.P., Eder C., Wright A.K., Frankenberger M., Marei A., Boghdadi G., Scherberich J., Ziegler-Heitbrock L. (2010). Standardized single-platform assay for human monocyte subpopulations: Lower CD14^+^CD16^++^ monocytes in females. Cytometry A.

[38] Perrot I., Deauvieau F., Massacrier C., Hughes N., Garrone P., Durand I., Demaria O., Viaud N., Gauthier L., Blery M. (2010). TLR3 and Rig-like receptor on myeloid dendritic cells and Rig-like receptor on human NK cells are both mandatory for production of IFN-γ in response to double-stranded RNA. J. Immunol..

[39] Ito T., Amakawa R., Kaisho T., Hemmi H., Tajima K., Uehira K., Ozaki Y., Tomizawa H., Akira S., Fukuhara S. (2002). Interferon-α and interleukin-12 are induced differentially by Toll-like receptor 7 ligands in human blood dendritic cell subsets. J. Exp. Med..

[40] Gorden K.B., Gorski K.S., Gibson S.J., Kedl R.M., Kieper W.C., Qiu X., Tomai M.A., Alkan S.S., Vasilakos J.P. (2005). Synthetic TLR agonists reveal functional differences between human TLR7 and TLR8. J. Immunol..

[41] Gbédandé K., Varani S., Ibitokou S., Houngbegnon P., Borgella S., Nouatin O., Ezinmegnon S., Adeothy A.L., Cottrell G., Massougbodji A. (2013). Malaria modifies neonatal and early-life toll-like receptor cytokine responses. Infect. Immun..

